# Associations Between Both HIV and Metabolic Comorbidity and Self-Reported Mpox Among Men Who Have Sex With Men: Multicenter Cross-Sectional Study

**DOI:** 10.2196/83450

**Published:** 2025-12-01

**Authors:** Yujie Liu, Xin Ge, Yinghuan Zhang, Yuxuan Wang, Meihui Zhang, Jianyu Chen, Gang Xu, Jiechen Zhang, Ying Wang, Yong Cai

**Affiliations:** 1 Public Health Research Center Tongren Hospital, Shanghai Jiao Tong University School of Medicine Shanghai China; 2 Shanghai Jiao Tong University School of Public Health Shanghai China; 3 College of Public Health Shanghai University of Medicine & Health Sciences Shanghai China; 4 Dermatology Department Tongren Hospital, Shanghai Jiao Tong University School of Medicine Shanghai China; 5 Center for Community Health Care China Hospital Development Institute, Shanghai Jiao Tong University Shanghai China

**Keywords:** HIV, metabolic comorbidity, mpox, men who have sex with men, additive interaction

## Abstract

**Background:**

Men who have sex with men (MSM) face a disproportionately high risk of mpox infection, and China has recently experienced a rapid increase in the reported cases. This population also has a high prevalence of HIV, which has been identified as a critical factor in understanding the vulnerability to mpox. In addition, metabolic diseases frequently co-occur with HIV and share immunometabolic pathways, raising concerns that they may interact to confer additional risk of mpox infection.

**Objective:**

This study examines the potential interaction between HIV and metabolic comorbidity in relation to self-reported mpox among MSM in China.

**Methods:**

A cross-sectional study was conducted among MSM aged 18 to 76 years from October 2023 to March 2024 in 6 representative provincial regions of China. Participants completed an anonymous questionnaire on HIV infection, metabolic diseases (hypertension, diabetes mellitus, and hyperlipidemia), and mpox infection. Metabolic comorbidity was defined as the presence of more than one of these conditions. Logistic regression models were used to examine associations, and additive and multiplicative interactions between HIV and metabolic comorbidity were assessed.

**Results:**

Of the 2403 MSM, 56 (2.33%) reported mpox, 199 (8.28%) reported HIV, and 325 (13.52%) reported at least one metabolic comorbidity (hypertension, diabetes, or hyperlipidemia). Both HIV (odds ratio [OR] 4.81, 95% CI 2.29-9.64) and metabolic comorbidity (OR 2.62, 95% CI 1.27-5.14) were associated with higher odds of mpox infection. A dose-response relationship was observed, with the odds of mpox increasing with the number of conditions (per-condition trend: OR 3.03, 95% CI 1.86-4.83). While multiplicative interaction was not statistically significant (interaction term=2.98, 95% CI 0.68-13.70; *P*=.15), additive interaction metrics suggested a possible excess association (relative excess risk due to interaction=10.80, 95% CI 1.21-37.52; attributable proportion due to interaction=0.74, 95% CI 0.07-0.87; synergy index=4.99, 95% CI 1.19-20.86). Compared to the participants without HIV or metabolic comorbidity, those with HIV and metabolic comorbidity had higher odds of mpox infection (OR 14.51, 95% CI 4.83-40.70).

**Conclusions:**

This study suggests that HIV and metabolic comorbidity were each associated with higher odds of self-reported mpox, and exploratory analyses indicated a possible additive interaction. Given the reliance on self-reported diagnoses and the cross-sectional design, the findings should be interpreted with caution due to reporting bias and reverse causation. Further studies are needed to confirm these associations and better understand the comprehensive health needs of MSM with co-occurring conditions.

## Introduction

Human mpox is a zoonotic disease caused by the monkeypox virus, originally endemic in Central and West African regions [1]. In May 2022, an outbreak of mpox occurred, characterized by a rapid increase in cases reported in previously nonendemic countries [2]. In response to the global outbreak of mpox, the World Health Organization (WHO) declared a public health emergency of international concern on July 23, 2022 [3]. In May 2023, the WHO announced that mpox no longer constituted a public health emergency of international concern [4]. In contrast to the global trend, the number of reported mpox cases in China has increased rapidly, with cases mainly concentrated in economically developed regions [5]. As of August 2025, China has reported 3531 cases and ranks among the 10 most affected countries [6].

Mpox primarily spreads through person-to-person transmission via close contact, including sexual activity. It has disproportionately affected men who have sex with men (MSM), who account for 85.4% of confirmed cases worldwide [6,7]. In China, 93.8% of mpox cases were identified within the MSM population [8]. Mpox is characterized by a skin rash or mucosal lesions that progress through several stages. These lesions can appear anywhere on the body, commonly affecting the face; palms; soles; and mucosal surfaces such as the mouth, genitals, and anus [7]. Vaccination has been shown to provide protective effects [9]. However, effective control in China remains challenging due to multiple factors, including difficulties in tracing infection sources, managing close contacts, and addressing stigma toward MSM [10,11].

MSM are also a vulnerable group to HIV infection. Worldwide, high prevalence rates of HIV have been consistently reported among MSM [12-14]. In China, consecutive cross-sectional surveys in the Jiangsu province documented prevalence estimates ranging from 8% to 9.8% between 2016 and 2020 [15]. During the global mpox outbreak, a substantial proportion of cases occurred in people living with HIV [16]. Studies have consistently documented overlap between mpox and HIV [17,18], which may reflect multiple, non–mutually exclusive factors such as overlapping sexual networks, differences in testing and health care access, and immune compromise [19]. These findings underscore HIV as a critical factor in understanding vulnerability to mpox within MSM populations.

Beyond this overlap, HIV is strongly associated with a broader range of chronic conditions, particularly metabolic comorbidity [20]. A recent meta-analysis estimated that the prevalence of metabolic syndrome among HIV-positive individuals was 25.3%, with significantly higher odds compared with their HIV-negative counterparts [21]. Even in those with normal body weight, HIV has been linked to poorer metabolic health, including unfavorable blood pressure and lipid and glucose profiles [22]. Such metabolic dysfunction is associated with chronic immune dysregulation and impaired antiviral responses, providing biologically plausible pathways through which metabolic comorbidity may influence susceptibility to viral infections [23].

Building on these observations, the co-occurrence of HIV and metabolic comorbidity may confer excess risk beyond individual effects. MSM are disproportionately exposed to adverse social conditions, and syndemic theory highlights that co-occurring health conditions can interact to generate excessive disease burdens [24]. Evidence from other infectious diseases supports this concern. In the context of COVID-19, coexistence of HIV and metabolic disorders has been shown to exacerbate disease severity [25]. Evidence from tuberculosis research demonstrates that co-occurrence of HIV and diabetes is associated with elevated mortality and measurable biological interaction, indicating synergistic rather than additive effects [26,27]. Although HIV and metabolic conditions have each been associated with mpox individually [18,28], whether their combined presence could pose additional mpox risk remains unclear.

This study aimed to address this knowledge gap by investigating the relationship between HIV, metabolic comorbidity, and mpox among MSM in 6 regions of China. Specifically, our hypothesis was that (1) HIV and metabolic comorbidity would be interrelated and individually associated with mpox infection and (2) HIV and metabolic comorbidity may interact in a way associated with a greater likelihood of mpox infection.

## Methods

### Participants and Procedure

A nationwide cross-sectional study was conducted from October 2023 to March 2024 in 6 geographically representative provincial regions of China: Shanghai, Guangdong, Xinjiang, Shaanxi, Yunnan, and Liaoning. Given the relatively hidden nature of the MSM population, recruitment was conducted in partnership with nongovernmental organizations (NGOs) that provide services and outreach to MSM.

Before the survey started, NGO staff received standardized training on study protocols, informed consent, and quality control procedures. Relying on their established networks and trust within MSM communities, the trained staff promoted the study and invited participation. Recruitment channels included both offline venues (eg, community events and in-person outreach) and web-based platforms (eg, WeChat groups). Potential participants were informed of the study’s objectives, procedures, and potential benefits before completing the questionnaire.

The inclusion criteria were as follows: (1) male and aged ≥18 years, (2) reporting of engagement in sexual behavior with men within the previous 6 months, and (3) regular residence in one of the 6 selected provincial regions (primarily residing locally for most of the previous 6 months). The exclusion criteria were as follows: (1) completion of the questionnaire in <300 seconds, (2) incorrect answers to quality control questions, and (3) IP address indicating a location outside the surveyed provincial regions. All eligible participants provided electronic informed consent and then completed the anonymous questionnaire via the Wenjuanxing web-based survey platform (Changsha Ranxing Information Technology Co).

The sample size was calculated using the prevalence of mpox as the primary outcome. Based on previous literature, the prevalence in MSM is approximately 5.6% [29]. Setting the allowable error (δ) at 0.20, the following sample size formula for cross-sectional surveys was applied:

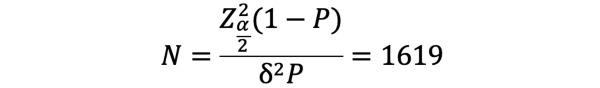



A sample size of 1619 was obtained. Considering a nonresponse rate of 15%, the final sample size for this survey was determined to be at least 1905 participants.

**Figure 1:**
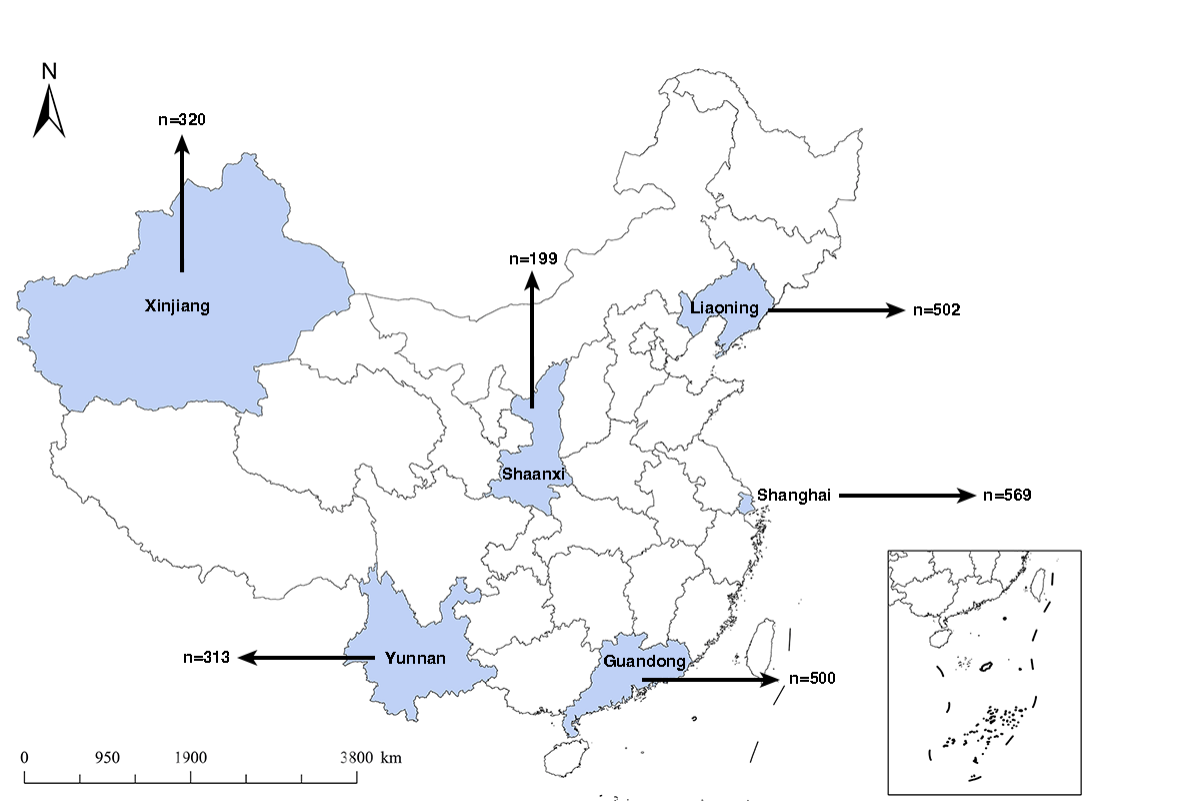
Distribution of the participants.

In this study, a total of 2481 questionnaires were collected, of which 2403 (96.86%) were valid. Figure 1 shows the geographic location and sample size of each surveyed area.

### Measurements

#### HIV Infection

Participants were asked the following: *What was your recent voluntary HIV testing (VHT) result*? A response of *positive* was considered a self-reported HIV infection.

#### Metabolic Comorbidity

This study primarily focused on 3 common metabolic diseases: hypertension, diabetes mellitus, and hyperlipidemia. Participants were asked whether they had been diagnosed with any of these conditions. The presence of at least one of these conditions was defined as having a metabolic comorbidity, consistent with a previous study [30]. Participants with metabolic comorbidity were referred to as the metabolically unhealthy group, whereas those without these conditions were referred to as the metabolically healthy group.

#### Self-Reported Mpox Infection

Participants were asked the following: *Have you been diagnosed with mpox by a doctor*? A response of *yes* was classified as a self-reported diagnosis of mpox.

#### Covariates

Sociodemographic characteristics included age, educational level (junior high school and lower, senior high school, and college and higher), marital status (single, married, and divorced or widowed), employment status (mental labor, manual labor, student, freelance work, and unemployed), duration of residence in the surveyed area (local, <5 years, and >5 years), and sexual orientation (homosexual and others).

Behavioral and clinical factors included the self-reported number of male sexual partners in the previous 6 months (1 or none, 2, and ≥3), condom use during anal intercourse with men in the previous 6 months (never, rarely, mostly, and always), and current use of antiretroviral therapy (yes or no).

### Statistical Analysis

Descriptive analyses were conducted to examine demographic characteristics and the prevalence of HIV and metabolic diseases. Chi-square tests were used to compare the distribution of categorical variables between the mpox infection and noninfection groups, whereas independent-sample 2-tailed *t* tests were used for continuous variables. Logistic regression models were constructed to investigate the bivariate associations among HIV, metabolic comorbidity, and mpox, as well as the associations between the number of health conditions and mpox infection while adjusting for demographic factors.

We examined the interaction between HIV and metabolic comorbidity in relation to mpox infection using logistic regression analyses [31], with 95% CIs estimated using the mover method as implemented in the *interactionR* R package (R Foundation for Statistical Computing) [32]. Interactions on both multiplicative and additive scales were assessed using interaction terms and indicator variables, including relative excess risk due to interaction (RERI), attributable proportion due to interaction (AP), and synergy index (SI) [33]. If the 95% CI of the AP or RERI contained 0 or the 95% CI of the SI contained 1, this indicated the absence of an interaction effect.

Sensitivity analyses were conducted to assess the robustness of our findings. Given the relatively small number of mpox cases, we performed Firth penalized logistic regression using the R package *logistf* to reduce small-sample bias and improve the stability of estimates [34]. In addition, to evaluate the potential impact of unmeasured confounding, we used the R package *EValue* to compute *E* values [35]. The *E* value quantifies the minimum strength of association that an unmeasured confounder would need to have with both the exposure and the outcome, conditional on the measured covariates, to fully explain the observed association.

### Ethical Considerations

All procedures performed in studies involving human participants were in accordance with the 1964 Declaration of Helsinki. The study protocol was approved by the Ethics Committee of Shanghai University of Medicine and Health Sciences (2023-MSMMPOX-22-310222197604080237). Written informed consent was obtained from all participants. Participants were informed that participation was voluntary and that they could withdraw from the study at any time without consequences. All data were anonymized before analysis, and no personally identifiable information was collected. Participants received a ¥50 (approximately US $7) gift card as compensation for their time.

## Results

### Characteristics of the Participants

Table 1 presents the characteristics of the 2403 participants aged 18 to 76 years, with a mean age of 30.59 (SD 8.02) years. Most of the participants were unmarried (2148/2403, 89.38%) and employed (1973/2403, 82.11%), whereas 78.57% (1888/2403) had an educational level of college or higher. A total of 38.95% (936/2403) of the participants were locals, and 24.84% (597/2403) had lived in the surveyed area for over 5 years. The sexual orientation of most participants (1887/2403, 78.53%) was homosexual. A total of 2.33% (56/2403) self-reported an mpox infection, and 8.28% (199/2403) self-reported an HIV infection. The prevalence rates of hypertension, diabetes mellitus, hyperlipidemia, and metabolic comorbidity were 6.45% (155/2403), 2.87% (69/2403), 9.24% (222/2403), and 13.52% (325/2403), respectively. The prevalence was 9.57% (230/2403) for participants with 1 metabolic condition and 3.95% (95/2403) for those with 2 or more conditions.

**Table 1:** Participant characteristics by self-reported mpox infection (N=2403).

			Total		Mpox infection				*P* value	
					No (n=2347)		Yes (n=56)			
Age (y), mean (SD)			30.59 (8.02)		30.66 (8.02)		27.38 (7.42)		.002	
**Educational level, n (%)**										.55
	Junior high school or lower	157 (6.53)		152 (6.48)		5 (9)				
	Senior high school	358 (14.90)		352 (15.00)		6 (11)				
	College or higher	1888 (78.57)		1843 (78.53)		45 (80)				
**Marital status, n (%)**										<.001
	Single	2035 (84.69)		1997 (85.09)		38 (68)				
	Married	255 (10.61)		238 (10.14)		17 (30)				
	Divorced or widowed	113 (4.70)		112 (4.77)		1 (2)				
**Employment status, n (%)**										.86
	Mental labor	386 (16.06)		376 (16.02)		10 (18)				
	Manual labor	774 (32.21)		757 (32.25)		17 (30)				
	Student	320 (13.32)		315 (13.42)		5 (9)				
	Freelance work	813 (33.83)		792 (33.75)		21 (38)				
	Unemployed	110 (4.58)		107 (4.56)		3 (5)				
**Duration of residence, n (%)**										.47
	Local	936 (38.95)		912 (38.86)		24 (43)				
	<5 y	870 (36.20)		848 (36.13)		22 (39)				
	≥5 y	597 (24.84)		587 (25.01)		10 (18)				
**Sexual orientation, n (%)**										.33
	Homosexual	1887 (78.53)		1846 (78.65)		41 (73)				
	Others	516 (21.47)		501 (21.35)		15 (27)				
**HIV, n (%)**										<.001
	No	2204 (91.72)		2164 (92.20)		40 (71)				
	Yes	199 (8.28)		183 (7.80)		16 (29)				
**Hypertension, n (%)**										.003
	No	2248 (93.55)		2201 (93.78)		47 (84)				
	Yes	155 (6.45)		146 (6.22)		9 (16)				
**Diabetes, n (%)**										.05
	No	2334 (97.13)		2282 (97.23)		52 (93)				
	Yes	69 (2.87)		65 (2.77)		4 (7)				
**Hyperlipidemia, n (%)**										.02
	No	2181 (90.76)		2135 (90.97)		46 (82)				
	Yes	222 (9.24)		212 (9.03)		10 (18)				
**Metabolic status, n (%)**										.003
	Healthy	2078 (86.48)		2037 (86.79)		41 (73)				
	Unhealthy	325 (13.52)		310 (13.21)		15 (27)				
**Number of sexual partners, n (%)**										.94
	1 or none	1206 (50.19)		1179 (50.23)		27 (48)				
	2	598 (24.89)		584 (24.88)		14 (25)				
	≥3	599 (24.93)		584 (24.88)		15 (27)				
**Condom use, n (%)**										<.001
	Never	126 (5.24)		123 (5.24)		3 (5)				
	Rarely	248 (10.32)		231 (9.84)		17 (30)				
	Mostly	634 (26.38)		607 (25.86)		27 (48)				
	Always	1395 (58.05)		1386 (59.05)		9 (16)				
**Antiretroviral therapy use, n (%)**										.06
	No	2356 (98.04)		2303 (98.13)		53 (95)				
	Yes	47 (1.96)		44 (1.87)		3 (5)				

Of the sociodemographic factors, age (*P*=.002) and marital status (*P*<.001) were associated with mpox infection. In addition, self-reported HIV (*P*<.001), hypertension (*P*=.003), hyperlipidemia (*P*=.02), and metabolic comorbidity (*P*=.003) were associated with higher odds of self-reported mpox infection.

### Bivariate Associations Among HIV, Metabolic Comorbidity, and Mpox Infection

Table S1 in Multimedia Appendix 1 presents the results of the adjusted logistic regression examining bivariate associations among HIV, metabolic comorbidity, and mpox infection. After adjusting for covariates, HIV was positively associated with metabolic comorbidity (odds ratio [OR] 2.49, 95% CI 1.63-3.74). Both HIV (OR 4.81, 95% CI 2.29-9.64) and metabolic comorbidity (OR 2.62, 95% CI 1.27-5.14) were associated with higher odds of mpox infection.

### Association Between Number of Health Conditions and Mpox Infection

Table 2 shows the results of the adjusted logistic regression examining the association between number of health conditions (ie, HIV and metabolic comorbidity) and mpox infection. After adjusting for covariates, an increase in the number of conditions was positively associated with mpox infection, suggesting a dose-response pattern (linear trend=3.03, 95% CI 1.86-4.83). Individuals with the simultaneous presence of HIV and metabolic comorbidity were associated with higher odds of mpox infection (OR 10.88, 95% CI 3.63-30.13).

**Table 2:** Association between number of health conditions (HIV and metabolic comorbidity) and self-reported mpox infection.

		Participants, n/N (%)	OR^a^ (95% CI)	*P* value
**Number of health conditions**				
	0	33/1927 (1.71)	Reference	Reference
	1	15/428 (3.5)	2.16 (1.08-4.15)	.02
	2	8/48 (17)	14.03 (4.64-39.59)	<.001
Linear trend		—^b^	3.03 (1.86-4.83)	<.001
**Co-occurring conditions**				
	No	48/2355 (2.04)	Reference	Reference
	Yes	8/48 (17)	10.88 (3.63-30.13)	<.001

^a^OR: odds ratio; adjusting for age, educational level, marital status, employment status, duration of residence, sexual orientation, number of sexual partners, condom use, and antiretroviral therapy use.

^b^Not applicable.

### Interaction Between HIV and Metabolic Comorbidity in Relation to Mpox Infection

Table 3 shows the results of the multivariable logistic regression examining interaction between HIV and metabolic comorbidity in relation to mpox infection. No statistically significant interaction was found between HIV and metabolic comorbidity (OR 2.98, 95% CI 0.68-13.70; *P*=.15). The RERI, AP, and SI for mpox infection resulting from HIV+metabolic comorbidity were 10.80 (95% CI 1.21-37.52), 0.74 (95% CI 0.07-0.87), and 4.99 (95% CI 1.19-20.86), respectively. The findings suggest a possible additive interaction between HIV and metabolic comorbidity. When examining each metabolic disease separately, HIV showed an interaction with hypertension in relation to mpox infection, whereas no interaction was observed with diabetes mellitus or hyperlipidemia (Table S2 in Multimedia Appendix 1).

**Table 3:** Interaction between HIV and metabolic comorbidity in relation to mpox infection.

			OR^a^ (95% CI)		*P* value
**Interaction model**					
	Unhealthy versus healthy	1.54 (0.61-3.83)		.36	
	HIV positive versus HIV negative	3.17 (1.32-7.60)		.009	
	Interaction term	2.98 (0.68-13.70)		.15	
Multiplicative scale			2.98 (0.67-13.21)		—^b^
RERI^c^			10.80 (1.21-37.52)		—
AP^d^			0.74 (0.07-0.87)		—
SI^e^			4.99 (1.19-20.86)		—

^a^OR: odds ratio, adjusting for age, educational level, marital status, employment status, duration of residence, sexual orientation, number of sexual partners, condom use, and antiretroviral therapy use.

^b^Not applicable.

^c^RERI: relative excess risk due to interaction.

^d^AP: attributable proportion due to interaction.

^e^SI: synergy index.

**Figure 2:**
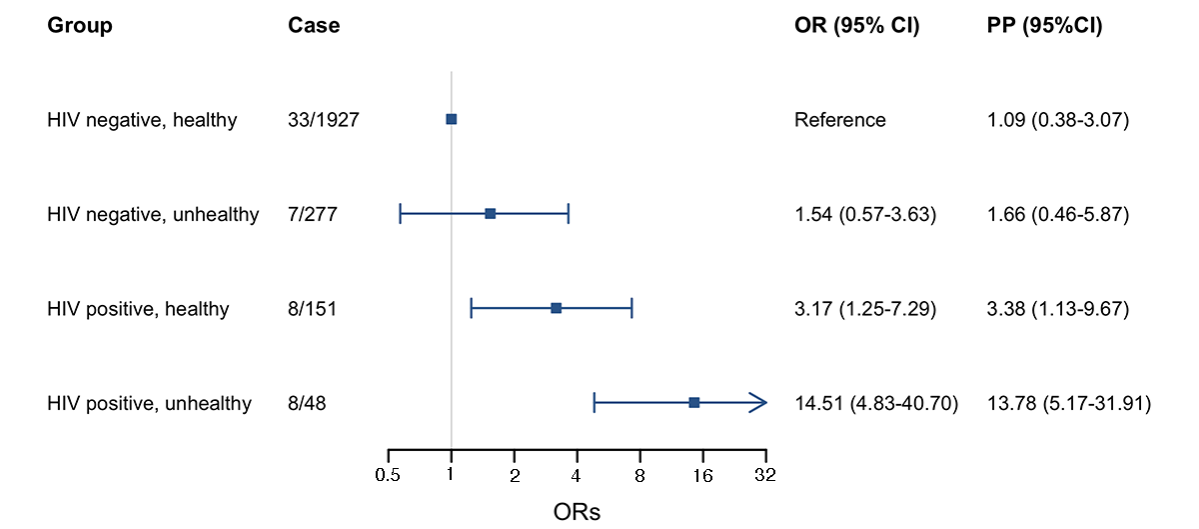
Odds ratios of self-reported mpox infection by HIV infection and metabolic status. OR: odds ratio; PP: predicted probabilities (%).

Figure 2 shows the relative odds of mpox infection by HIV infection and metabolic status. Compared to individuals without self-reported HIV and who were metabolically healthy, those with self-reported HIV but who were metabolically healthy (OR 3.17, 95% CI 1.25-7.29) and those with both self-reported HIV and metabolic comorbidity (OR 14.51, 95% CI 4.83-40.70) had higher odds of self-reported mpox infection.

### Sensitivity Analysis

The estimates of the additive interaction between HIV and metabolic comorbidity obtained using the Firth penalized likelihood logistic regression were consistent with those from the main analysis. Specifically, the RERI was 9.56 (95% CI 1.16-30.90), the AP was 0.71 (95% CI 0.08-0.86), and the SI was 4.38 (95% CI 1.20-15.94), supporting a possible additive interaction. Penalized regression models assessing mpox infection by HIV infection and metabolic status produced results that were in line with the primary findings (Table S3 in Multimedia Appendix 1).

In the quantitative bias analysis, the group with self-reported HIV but who was metabolically healthy yielded an *E* value of 5.79 (lower 95% CI bound=1.81), suggesting that an unmeasured confounder would need to be associated with both exposure and outcome by an OR of at least 5.79 to fully account for the observed association. For the group with both self-reported HIV and metabolic comorbidity, the *E* value was 28.51 (lower 95% CI bound=9.13), indicating that only a strong unmeasured confounder could explain the observed association.

## Discussion

### Principal Findings

This study offers valuable insights into the associations among HIV, metabolic comorbidity, and self-reported mpox. In our sample of MSM in China, we observed a clustering of HIV and metabolic comorbidity, both of which were individually associated with mpox infection. In addition, our exploratory analyses suggested a possible additive interaction between HIV and metabolic comorbidity in relation to mpox infection.

MSM, as a sexual minority population, often face social, economic, and environmental disadvantages, leading to a higher prevalence of adverse health conditions [36]. In this study among MSM, the prevalence rates of HIV and metabolic comorbidity were 8.28% (199/2403) and 13.52% (325/2403), respectively, with a positive correlation observed between the 2 health conditions. Consistent with previous findings that metabolic diseases tend to occur earlier in people living with HIV [20], we found that 24.1% (48/199) of MSM who were infected with HIV simultaneously exhibited metabolic comorbidity. The underlying factors contributing to these comorbidities are multifaceted and include antiretroviral therapy use, unhealthy lifestyle behaviors (ie, smoking and alcohol use), and chronic inflammation despite suppressed HIV replication [37].

Existing studies among MSM have predominantly examined the interplay of psychological, social, and structural factors using the syndemic framework [38-40], whereas relatively few have focused on HIV and co-occurring physical health conditions [41,42]. This study provides exploratory observations on the association between HIV and metabolic comorbidity in relation to mpox infection among MSM. While previous research has documented associations between both HIV and metabolic diseases and mpox infection individually [18,28], our analysis, through calculation of additive interaction measures (RERI, AP, and SI), suggests a previously unrecognized possibility of interaction between HIV and metabolic comorbidity. These findings suggest that MSM with both HIV and metabolic comorbidity may warrant further investigation in future research and consideration in the design of integrated health services.

In this study, we identified 2.33% (56/2403) of cases of mpox infection among MSM in China. This prevalence rate is notably higher than the 0.73% reported in a previous nationwide survey conducted in early August 2023 [43], primarily because a substantial number of confirmed mpox cases in China were reported after that [8]. In addition, despite the clear clustering of HIV with metabolic comorbidity, the prevalence rates of each metabolic condition, namely, hypertension (155/2403, 6.45%), diabetes mellitus (69/2403, 2.87%), and hyperlipidemia (222/2403, 9.24%), were relatively low in our sample. This may explain the nonsignificant interaction observed between HIV and each separate metabolic disease in this study.

### Implications

Our findings suggest that HIV and metabolic health issues tend to cluster and interact, and exploratory analyses suggested a possible additive interaction in relation to mpox infection. Understanding the individual and contextual factors underlying these interactions—such as substance use, living conditions, and social networks—will be important for informing future research [41,44]. Although this study was conducted in China, the results may offer insights relevant to other low- and middle-income countries, where HIV and noncommunicable diseases are highly prevalent and health systems often underresourced [45,46]. Strengthening resilient health systems capable of delivering integrated care is crucial for advancing universal health coverage [47]. Previous research has shown that interventions focusing solely on individual conditions such as HIV have limited effectiveness in improving overall health [48,49]. Building on these lessons, global experiences from infectious disease emphasize the need for systematic health system redesign and stronger international collaboration [50]. In this context, MSM with multiple conditions may represent a subgroup warranting attention in future comprehensive health services. However, the design and evaluation of such interventions should be based on stronger empirical evidence from future studies.

### Limitations

This study has several limitations. First, HIV status, mpox status, and metabolic comorbidity were self-reported, which may have introduced misclassification and reporting bias. Given that mpox-related stigma among MSM can discourage care-seeking behaviors [51], underreporting of previous diagnoses is plausible. In epidemiological studies, nondifferential misclassification due to underreporting typically reduces sensitivity while maintaining high specificity, which tends to bias effect estimates toward the null [52]. Therefore, our findings are likely conservative and should be interpreted as exploratory. Second, the sample was recruited through NGOs and web-based platforms, potentially overrepresenting MSM who are more engaged with health services and limiting generalizability to hidden or higher-risk subgroups. Third, the cross-sectional design precludes causal inference. As a result, the temporal sequence among HIV, metabolic comorbidity, and self-reported mpox cannot be established, and reverse causation remains possible.

Fourth, the number of mpox cases was relatively small, which limited statistical power and precision, especially for interaction analyses. The wide CIs for the interaction terms reflect this instability. Although we conducted sensitivity analyses using the Firth penalized likelihood logistic regression to mitigate sparse-data bias and found consistent results, the findings should still be interpreted as exploratory. Fifth, our study focused on 3 common metabolic diseases—hypertension, diabetes mellitus, and hyperlipidemia—potentially overlooking other important conditions such as obesity. Finally, while we adjusted for several behavioral and clinical covariates, other relevant factors such as sex work, history of other sexually transmitted diseases, viral load, and vaccination status were not collected, which may have led to residual confounding. However, our quantitative bias analysis indicated that only unmeasured confounders with very strong associations with both exposure and outcome could fully explain the observed results, suggesting that the main findings are relatively robust. Taken together, these limitations highlight the need for future studies with larger and more representative samples; objective diagnostic measures; and more comprehensive assessments of biomedical, behavioral, and contextual factors.

### Conclusions

Our findings suggest a potential clustering and additive interaction of HIV and metabolic comorbidity in relation to self-reported mpox infection among MSM. These exploratory results highlight the importance of considering co-occurring health conditions among MSM and may offer insights for similar populations in other settings with high burdens of HIV and noncommunicable diseases.

## Data Availability

The datasets generated or analyzed during this study are not publicly available due to privacy or ethical restrictions but are available from the corresponding author on reasonable request.

## Appendix

Multimedia Appendix 1 Additional tabulated results.

Multimedia Appendix 2 STROBE checklist.

## Abbreviations

AP: attributable proportion due to interaction
MSM: men who have sex with men
NGO: nongovernmental organization
OR: odds ratio
RERI: relative excess risk due to interaction
SI: synergy index
STROBE: Strengthening the Reporting of Observational Studies in Epidemiology
WHO: World Health Organization
