# Supplementary material for: Associations Between Both HIV and Metabolic Comorbidity and Self-Reported Mpox Among Men Who Have Sex With Men: Multicenter Cross-Sectional Study
Source: JMIR Public Health Surveill. 2025 Dec 1;11:e83450. doi: 10.2196/83450 (PMC12706452; doi:10.2196/83450)
Supplement: Multimedia Appendix 1 [file publichealth_v11i1e83450_app1.docx]

**Table S1 Bivariate associations between HIV infection, metabolic comorbidity, and mpox infection**

|  | **Metabolic comorbidity** | | **Mpox infection** | |
| --- | --- | --- | --- | --- |
|  | OR (95% CI) | p value | OR (95% CI) | p value |
| **HIV infection** | 2.49 (1.63 – 3.74) | <.001 | 4.81 (2.29 – 9.64) | <.001 |
| **Metabolic comorbidity** |  |  | 2.62 (1.27 – 5.14) | 0.006 |

OR: odds ratio, adjusting for age, education level, marital status, employment, length of residence, sexual orientation, number of sexual partners, condom use, and antiretroviral therapy use.

CI: confidence interval

**Table S2 Interaction between HIV infection and individual metabolic disease in relation to mpox infection**

|  | Mpox Infection | |
| --- | --- | --- |
|  | OR (95% CI) | p value |
| Hypertension | | |
| HTN (+) versus HTN (-) | 1.13 (0.31, 4.14) | 0.85 |
| HIV (+) versus HIV (-) | 3.63 (1.64, 8.03) | 0.001 |
| Interaction term | 4.59 (0.74 – 33.21) | 0.11 |
| Multiplicative scale | 4.59 (0.71 – 29.82) | |
| RERI | 15.12 (1.32 – 66.43) | |
| AP | 0.80 (0.04 – 0.89) | |
| SI | 6.47 (1.25 – 33.42) | |
| Diabetes | | |
| DM (+) versus DM (-) | 0.93 (0.11, 7.67) | 0.94 |
| HIV (+) versus HIV (-) | 4.06 (1.85, 8.87) | <.001 |
| Interaction term | 3.24 (0.27 – 83.47) | 0.38 |
| Multiplicative scale | 3.24 (0.23 – 46.47) | |
| RERI | 8.21 (-3.98 – 50.97) | |
| AP | 0.67 (-1.25 – 0.82) | |
| SI | 3.75 (0.52 – 27.07) | |
| Hyperlipidemia | | |
| HLD (+) versus HLD (-) | 2.26 (0.81, 6.32) | 0.12 |
| HIV (+) versus HIV (-) | 4.21 (1.90, 9.35) | <.001 |
| Interaction term | 1.16 (0.22 – 6.11) | 0.86 |
| Multiplicative scale | 1.16 (0.23 – 5.96) | |
| RERI | 5.56 (-3.60 – 31.50) | |
| AP | 0.50 (-1.02 – 0.75) | |
| SI | 2.24 (0.52 – 9.67) | |

OR: odds ratio, adjusting for age, education level, marital status, employment, length of residence, sexual orientation, number of sexual partners, condom use, and antiretroviral therapy use.

CI: confidence interval; RERI: relative excess risk due to interaction; AP: attributable proportion due to interaction; SI: synergy index.

**Table S3. Firth’s penalized likelihood logistic regression of self-reported mpox infection by HIV infection and metabolic status**

| Group | Mpox Infection | |
| --- | --- | --- |
|  | OR (95% CI) | p value |
| HIV (-), healthy | reference | – |
| HIV (-), unhealthy | 1.59 (0.61 – 3.66) | 0.32 |
| HIV (+), healthy | 3.24 (1.32 – 7.20) | 0.019 |
| HIV (+), unhealthy | 13.39 (4.67 – 35.97) | <.001 |

OR: odds ratio, adjusting for age, education level, marital status, employment, length of residence, sexual orientation, number of sexual partners, condom use, and antiretroviral therapy use.

CI: confidence interval
